# Comprehensive evaluation of surface water quality: heavy metals, speciation, and human health risks in an industrial region of West Bengal, India

**DOI:** 10.1007/s10661-026-15424-9

**Published:** 2026-05-23

**Authors:** Gourav Mondal, Riddhi Basu, Sumit Kumar, Shreya Chakraborty, Ambika Barman, Kasturi Charan, Jajati Mandal, Pradip Bhattacharyya

**Affiliations:** 1 Agricultural and Ecological Research Unit, Indian Statistical Institute, Giridih, Jharkhand 815301 India; 2 School of Sciences, University of Salford, Engineering & Environment, Manchester, M5 4WT UK

**Keywords:** Heavy metals, Water pollution, Health risk assessment, Carcinogenic risk

## Abstract

This study evaluates the hydrogeochemical characteristics, heavy metal (HM) contamination, irrigation suitability, and associated human health risks of surface water in an industrial area affected by dye-manufacturing activities in West Bengal, India. A total of 30 surface water collected in triplicate were analysed. Major ions were determined using standard American Public Health Association (APHA)  methods, HMs were quantified using atomic absorption spectrometry (AAS), and aqueous speciation was modelled using Visual MINTEQ. The water was slightly alkaline, with pH values ranging from 7.18 to 8.92 (mean 7.79±0.41). Extremely high salinity was observed, as electrical conductivity ranged from 2500 to 21,000 μS cm⁻¹ (mean 11,300±4272 μS/cm), while total dissolved solids varied between 1500 and 12,600 mg/L (mean 6780 ± 2563 mg/L), exceeding World Health Organization (WHO) guideline values. Major cations followed the order Ca²⁺ > Mg²⁺ > Na⁺ > K⁺, whereas dominant anions were HCO_3_⁻ > Cl⁻ > SO_4_^2^⁻, reflecting combined geogenic and anthropogenic influences. Mean concentrations of heavy metals were elevated, with values of Cr (0.97 mg/L), Pb (0.23 mg/L), Ni (0.19 mg/L), Cu (0.06 mg/L), and Cd (0.004 mg/L). Except for Cu, all metals exceeded WHO permissible limits, indicating substantial contamination. Pollution indices (Pi) confirmed severe degradation, with Pi > 1 for Pb, Ni, Cr, and Cd. High average values of C_d_ (48.89), Heavy Metal Evaluation Index (HEI), 53.65, and Heavy Metal Pollution Index (HPI), 338.73, indicated high to extreme pollution, while Water Pollution Index (WPI) values (1.51–5.27) classified all samples as severely polluted. Speciation modelling showed Cd predominantly occurred as Cd^2+^ (52.35%) and CdCl^+^ (32.10%), suggesting high mobility. Lead (Pb) was predominantly present as PbCO_3_(aq) (39.47%) and PbHCO_3_^+^ (31.31%), Ni largely as Ni^2+^ (63.15%), and Cr(VI) mainly as CrO_4_^2^⁻. Copper was largely present as CuCO_3_(aq) (67.94%). Irrigation indices indicated suitability, although overall water quality remained poor. Human health risk assessment identified Pb and Cr as posing non-carcinogenic risks, while Cr presenting the highest carcinogenic risk, particularly for children (CR > 1 × 10⁻^4^). Overall, the findings highlight severe surface water degradation and underscore the urgent need for remediation measures and stricter regulatory intervention.

**Supplementary Information:**

The online version contains supplementary material available at 10.1007/s10661-026-15424-9.

## Introduction

Water is the most crucial requisite for all life forms (Westall & Brack, 2018). It is additionally an irreplaceable natural resource that influences the socioeconomic growth as well as the sustainable development of a nation. Despite that, water is the most poorly managed resource and has been facing serious threats, especially due to anthropogenic activities (Akhtar et al., 2021; Bashir et al., 2020; Ejiohuo et al., 2025). Especially in areas supporting rapid industrialisation, water contamination due to heavy metals (HMs) has served to be a crucial environmental as well as public health problem (Ali et al., 2021). Surface water bodies, such as lakes, rivers, and wetlands across the world, are getting increasingly contaminated with toxic heavy metals such as lead (Pb), chromium (Cr), nickel (Ni), cadmium (Cd), copper (Cu), and zinc (Zn). The sources of these metals generally are industrial discharges, mining, smelting, electroplating, tanneries, e-waste recycling, agricultural runoff, and untreated urban wastewater (Mustapha et al., 2025). On a global level, certain regions of South and Southeast Asia, China, Sub-Saharan Africa, Eastern Europe, and South America’s mining belts have been showing the highest levels of contamination (Li et al., 2020). The water bodies in these regions report metal concentrations exceeding WHO and USEPA safety limits find its way in sediments, where they act as long-term sources of pollution, releasing back into the water column during flooding, drought, or any other seasonal disturbances (Lapworth et al., 2022). In India, surface water pollution reports are again numerous. The Ganga basin has been reported to have high heavy metal contamination, especially from anthropogenic sources, significantly degrading water quality (Shukla et al., 2021). Another recent study (Singh et al., 2025) on the Hindon River (Muzaffarnagar district) has reported contamination, and seasonal variation in pollution shock by heavy metals such as Cd, Cr, Fe, Mn, Zn, Cu, and Pb. Additionally, a study of the Buckingham Canal (Chennai, Tamil Nadu) reported that concentrations of Cr, Cu, Fe, Pb, and Zn exceeded permissible limits, suggested potential ecological disruption and human health concerns arising from their accumulation in the aquatic food chain (Kumar et al., 2018). On a broader scale, a government/monitoring-report-based study (CWC, 2024) indicated that 81 Indian rivers or tributaries have “extremely high” concentrations of one or more toxic heavy metals. Lack of proper monitoring and regulatory policies in developing nations, India being one of them, has led to steady allowance of dumping of untreated or inadequately treated industrial discharges in surface water bodies, which ultimately finds its way into the food chain, while subsequently deteriorating the overall quality of the water body (Tariq & Mushtaq, 2023).

Heavy metals are priority pollutants among the huge number of toxicants released in water due to their high bioaccumulation capacity and persistent nature (Mustapha et al., 2025). Textile and dye industries, along with bringing upon economic stability, often lead to extensive HM pollution. HMs such as lead (Pb), zinc (Zn), chromium (Cr), and copper (Cu) are commonly employed as catalysts in fibre production and as essential oxidising agents in various textile wet-processing operations (Catarino et al., 2025). Additionally, the dye industry is also a major source because many synthetic dyes and mordants contain metals such as Cr, Cu, Zn, and Pb (Manian et al., 2016). These metals are released through untreated or partially treated effluents, contaminating soil and water bodies. In aquatic bodies, such toxic heavy metals do not degrade for a long time instead leads to accumulation into sediments and the biota thereby posing a long-term risk to ecology and human health (Mustapha et al., 2025). Chronic exposure to these toxic metals has often been associated with carcinogenic effects, neurological disorders, renal dysfunction, and impaired growth in both humans and aquatic organisms (Ohiagu et al., 2022). The effect of such contamination is especially grave in densely populated urban and peri-urban regions where surface water bodies serve both as domestic resources as well as drainage outlets. The situation further intensifies when industrial effluents also find its way in small canals, which lack the system of dilution like that of large river systems and thereby result in local pollution hotspots (Gomes et al., 2019).

West Bengal has seen a rapid expansion of unregulated, small- and medium-scale industries, especially dye, textile, and electroplating industries or metal finishing units, which have often contributed to concerns relating to surface water quality (Gupta, 2019). The Noai canal in Ghola, Sodepur, is another vulnerable aquatic ecosystem receiving continuous anthropogenic inputs and thus deserves quality analysis. Considering the canal is surrounded by residential areas, there lies a high chance of direct human exposure through routes of domestic usage, dermal contact, or accidental ingestion of the canal water. Thus, a comprehensive evaluation of the water quality including the physiochemistry, quality index estimation, and health risk analysis was attempted from this canal water. Noai canal has not been previously studied and thus the estimated risk associated with the position of the canal the study can serve utterly important in management and protection of the water body. The core hypothesis guiding this work is that the Noai canal shows degraded water quality, elevated heavy metal levels, and measurable human health risks resulting from ongoing discharges. Therefore, this study aims to elucidate the water quality of Noai canal considering the following objectives: (i) to assess the physicochemical properties of the canal water, including major ions and heavy metal concentrations; (ii) to evaluate overall water quality and irrigation suitability using established water quality indices; (iii) to examine spatial patterns of heavy metal distribution with geostatistical methods; (iv) to estimate the potential health risks associated with heavy metal exposure. This integrated approach is intended to build a clear and actionable understanding of the contamination scenario, supporting more effective management and protection of both the canal and the surrounding community.

## Materials and methods

### Geomorphological description of the study site

The study region, Ghola, is in North 24 Parganas district under the Barrackpore subdivision, West Bengal, India (22° 41′ 47″ N, 88° 24′ 32″ E). The area covers 19.43 km^2^ and comprises 355 wards with a population over 0.4 million (Chaudhri et al., 2015). The landscape of the area is shaped by its location on the Ganga-Bhagirathi delta’s lower plain, encompassing recent sediments deposited by the river during the quaternary period. This region experiences a scorching, humid summer which spans from March to June with temperature exceeding 38 °C. The average annual rainfall in the area is around 150–200 cm, 75% of which occurs during the monsoon. Winters are short-lived and mild with temperatures ranging from 13 to 26 °C (Roy & Roy, 2021). This area features both residential and industrial sites. The studied region possesses a diverse industrial landscape with chemical manufacturing, food processing, and textile industries. However, urban sprawl and rapid industrial effluent discharge have raised concern about nearby waterbody contamination particularly from HM pollution, questioning human well-being and environmental sustainability.

### Collection of water samples

**Fig. 1:**
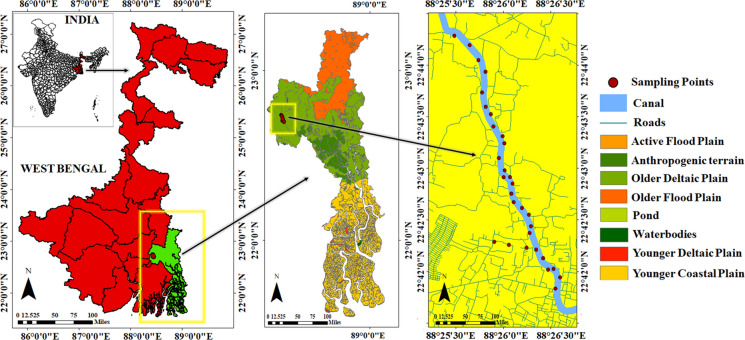
Geological map of the study area showing water sampling locations

A total of 30 water samples including three biological replicates were collected before monsoons from the Noai canal in Ghola, Sodepur, North 24 Parganas district, West Bengal, India (Fig. 1) with precise GPS coordinates. High-density polyethylene (HDPE) bottles were used for the sample collection. The samples were preserved by acidification using 0.1% (v/v) concentrated nitric acid and transported to the laboratory under standard procedures established by APHA (2012) in ice boxes. All samples were kept at 4 °C prior to being analysed.

### Estimation of major ionic abundance

The physicochemical characteristics and major ion abundance of the collected water samples (*n* = 30) were determined using standardised laboratory protocols (APHA, 2012). pH and electrical conductivity (EC) of the water samples were determined by adopting standard protocol in a pH and conductometer meter of Systronics India (Kumar et al., 2025). Available potassium (K^+^), sodium (Na^+^), and calcium (Ca^2+^) were measured in a flame photometer (1027 Systronics, India), while magnesium (Mg^2+^) was analysed using an atomic absorption spectrophotometer (AAS-816, Systronics). Sulphate (SO_4_^2−^) was estimated in type 117 UV–vis spectrophotometer. The concentration of phosphate (PO_4_^2^⁻) was detected using the ammonium molybdate method, nitrate-nitrogen (NO_3_-N) was measured by spectrophotometric method, ammoniacal nitrogen in water was measured using the Kjeldahl method, biological oxygen demand (BOD) was estimated following Winkler’s procedure, chemical oxygen demand (COD) was determined using the chromate method, while chloride (Cl⁻) and bicarbonate (HCO_3_⁻) ions were quantified through titrimetric analysis. Total dissolved solids (TDS) and total hardness (TH) were calculated using formulas from previous literature (Mondal et al., 2025).

1 $$\mathrm{T}\mathrm{D}\mathrm{S} = \mathrm{E}\mathrm{C} \times 0.65$$

2 $$\mathrm{TH}\:=\:2.5\:\times\:\mathrm{Ca}^{2+}\:+\:4.1\:\times\:\mathrm{Mg}^{2+}$$

The study used an ion-selective probe (Thermoscientific, USA) to measure fluoride (F⁻) levels (Gantait et al., 2022). To find the concentrations of the chosen heavy metals (HMs) (Pb, Ni, Cr, Cu, and Cd), an AAS (Model-816, Systronics) was used. As part of quality control procedures, a blank extract and certified reference material (SRM 2710) were utilised to ensure accuracy and detect any possible contamination (Supplementary Table 1). The ionic balance equation (IBE) was used to check the precision and minimise errors of all ionic measurements (Eq. 3). The calculated values for each sample fell within the generally accepted limit of ± 10%, and the measurements were accepted as accurate and reliable.

3 $$\frac{IBE}{CBE}=\frac{\sum cations-\sum anions}{\sum cations-\sum anions}\times100$$

### Measuring water quality through different indices

#### Single factor pollution index (P_i_)

The Single Factor Index assesses overall water quality by comparing measured pollutant concentrations with standard limits. It helps to identify key pollutants and their potential risks. The pollution index for each parameter was determined using the following Eq. (4):

4 $${P}_{i}= \frac{{C}_{i}}{{S}_{i}}$$

where *P*_*i*_ is the pollution index for the *i*th parameter, *C*_*i*_ is the observed concentration (mg/L), and *S*_*i*_ is the corresponding standard value (mg/L). Based on *P*_*i*_, water quality is categorised as follows: *P*_*i*_ < 1 (clean), 1–2 (slightly polluted), 2–3 (lightly polluted), 3–5 (moderately polluted), and *P*_*i*_ > 5 (heavily polluted) (Linjin et al., 2021).

#### Degree of Contamination (C_d_)

Li et al. (2015) assessed degree of heavy metal contamination (*C*_*d*_) in environmental samples using the contamination factor. In this approach, the contamination factor (*Cf*_*i*_) for each metal is determined by comparing its measured concentration (*M*_*i*_) with the corresponding standard or background value (*S*_*i*_), as expressed in Eqs. (5) and (6):

5 $${C}_{d}=\sum\nolimits_{i=1}^{n}{Cf}_{i}$$

6 $${Cf}_{i}= \frac{{M}_{i}}{{S}_{i}}-1$$

Based on the calculated *C*_*d*_ values, contamination levels are classified as low (*C*_*d*_ ≤ 1), moderate (1 < *C*_*d*_ < 3), or high (*C*_*d*_ > 3) (Edet & Offiong, 2002).

#### Heavy metal evaluation index (HEI) and heavy metal pollution index (HPI)

The Heavy Metal Evaluation Index (HEI) offers an overall assessment of water quality by considering the combined effects of heavy metals on human health. It is calculated as the sum of the ratios between the measured concentration of each metal (*Cᵢ*) and its respective maximum allowable concentration (MAC):

7 $$\mathrm{H}\mathrm{E}\mathrm{I}=\sum\nolimits_{i=1}^{n}\frac{{C}_{i}}{{\mathrm{M}\mathrm{A}\mathrm{C}}_{i}}$$

Based on HEI values, water quality is categorised as low (HEI < 10), medium (10–20), or high (> 20) contamination levels. The Heavy Metal Pollution Index (HPI) was proposed to access the water quality based on HM contamination. Each HMs were assigned an arbitrary value of 0 to using equations proposed by Giri and Singh (2019) to determine the overall pollution index for water samples. Water quality was classified into five categories: excellent (HPI < 25); good (26–50); poor (51–75); very poor (76–100); and unsuitable for consumption (HPI > 100) (Mohan et al., 1996).

#### Determination of water pollution index (WPI)

The Water Pollution Index (WPI) serves as a flexible and logical tool for assessing overall water quality by integrating multiple chemical parameters into a single representative value (Horton, 1965; Hossain & Patra, 2020a, b). In this study, pH, total dissolved solids (TDS), total hardness (TH), calcium (Ca^2+^), magnesium (Mg^2+^), sodium (Na^+^), potassium (K^+^), bicarbonate (HCO_3_⁻), sulphate (SO_4_^2^⁻), chloride (Cl⁻), nitrate (NO_3_⁻), phosphate (PO_4_^3−^), fluoride (F⁻), lead (Pb), nickel (Ni), chromium (Cr), copper (Cu), and cadmium (Cd) were used to compute the WPI. This approach consists of two major steps. The first step involves the estimation of pollution load (PL*ᵢ*) as shown in Eq. (8):

8 $${\mathrm{P}\mathrm{L}}_{i}=1+ \frac{{C}_{i}-{S}_{i}}{{S}_{i}}$$

where PL*ᵢ* is the pollution load of the *i*th parameter, *Cᵢ* represents the observed concentration, and *Sᵢ* denotes the maximum desirable concentration for drinking water. When pH < 7, the pollution load is obtained using Eq. (9):

9 $${\mathrm{P}\mathrm{L}}_{i}=1+ \frac{{C}_{i}-7}{{S}_{a}-7}$$

where *Sₐ* (6.5) is the minimum acceptable pH limit for potable water. When pH > 7, the pollution load is estimated using Eq. (10):

10 $${\mathrm{P}\mathrm{L}}_{i}=1+ \frac{{C}_{i}-7}{{S}_{b}-7}$$

where *S*_*b*_ (8.5) is the upper permissible limit for drinking water quality. In the second step, the Water Pollution Index is determined using Eq. (11):

11 $$\mathrm{W}\mathrm{P}\mathrm{I}=\frac{1}{n}\sum\nolimits_{i=1}^{n}{\mathrm{P}\mathrm{L}}_{i}$$

where *n* is the total number of parameters considered. It classifies water into four classes, i.e. excellent (WPI < 0.5), good (WPI 0.5–0.75), moderately polluted (WPI 0.75–1).

#### Irrigation indices

Different irrigation indices like sodium absorption ratio (SAR), Kelly’s ratio (KR), and percent sodium (%Na) were calculated using formulas adopted by Rawat et al. (2018). A high sodium percentage value generally reflects alkalinity hazards associated with reduced soil infiltration and drainage and might exhibit a negative impact on soil heath due to precipitation of Ca and Mg. A significantly high KR value is also associated with alkalinity hazard in soil (Gantait et al., 2022).

### Health risk assessment

#### Non-carcinogenic risk

The surface water is vulnerable to HM contamination. Human body can be exposed to surface water by oral, inhalation, and dermal route (Ganvir & Papadkar, 2022). The study followed the US EPA’s health risk assessment model, evaluating risks from environmental pollutants in two categories: carcinogenic risks (CR) and non-carcinogenic risks (NCR) (USEPA, 2018). Hazard quotient (HQ) was determined based on the exposure pathway (ingestion and dermal) and the target groups. This research focused considered two age group, i.e. children and adults. The chronic daily intake (CDI) of the studied HM (Cd, Ni, Pb, Cr, and Cu) was assessed using following equation.

12 $${\mathrm{C}\mathrm{h}\mathrm{r}\mathrm{o}\mathrm{n}\mathrm{i}\mathrm{c}\, \mathrm{D}\mathrm{a}\mathrm{i}\mathrm{l}\mathrm{y} \,\mathrm{I}\mathrm{n}\mathrm{t}\mathrm{a}\mathrm{k}\mathrm{e}\,\left(\mathrm{C}\mathrm{D}\mathrm{I}\right)}_{\mathrm{i}\mathrm{n}\mathrm{g}\mathrm{e}\mathrm{s}\mathrm{t}\mathrm{i}\mathrm{o}\mathrm{n}}=\frac{C*ED*IR*EF}{BW*AT}$$

13 $${\mathrm{C}\mathrm{h}\mathrm{r}\mathrm{o}\mathrm{n}\mathrm{i}\mathrm{c}\,\mathrm{D}\mathrm{a}\mathrm{i}\mathrm{l}\mathrm{y} \,\mathrm{I}\mathrm{n}\mathrm{t}\mathrm{a}\mathrm{k}\mathrm{e}\,\left(\mathrm{C}\mathrm{D}\mathrm{I}\right)}_{\mathrm{d}\mathrm{e}\mathrm{r}\mathrm{m}\mathrm{a}\mathrm{l}}= \frac{C\times SA\times AF\times {ABS}_{\mathrm{d}\mathrm{e}\mathrm{r}\mathrm{m}\mathrm{a}\mathrm{l}}\times EF\times ED}{BW\times AT}\times {10}^{-6}$$

In the above-mentioned equation, *C* denotes HM concentration in the water sample. *ED* stands for exposure duration (children = 6 years, adults = 24 years). *IR* symbolises daily ingestion rate (children = 1 L/day, adults = 2 L/day). 365 days is the exposure frequency (*EF*), and *BW* stands for body weight which is 57 kg for adults and 18.7 kg for children. *AT* represents average lifetime (adults = 8760 days, children = 2190 days), *AF* represents skin adherence factor (adults = 0.07 mg/cm^2^-event, children = 0.2 mg/cm^2^-event), *ABS*_dermal_ denotes dermal absorption fraction of HMs (0.001), and 10⁻^6^ factor converts the water concentration unit from mg/L to kg/L.

Non-carcinogenic risk was related to hazard quotient (HQ) and it was formulated from Eq. (14):

14 $$HQ= \frac{\mathrm{C}\mathrm{D}\mathrm{I}}{\mathrm{R}\mathrm{e}\mathrm{f}\mathrm{e}\mathrm{r}\mathrm{e}\mathrm{n}\mathrm{c}\mathrm{e} \,\mathrm{d}\mathrm{o}\mathrm{s}\mathrm{e} \,({R}_{f} D)}$$

HQ values were calculated separately for children and adults. A higher HQ value (> 1) reflects a non-carcinogenic health risk for human and should not recommend for consumption and for daily use (Oni et al., 2022).

#### Carcinogenic risk

Cancer risk (CR) states the possibility of developing cancer after the exposure of potential carcinogen and formulated using following Eq. (15)

15 $$\mathrm{C}\mathrm{a}\mathrm{r}\mathrm{c}\mathrm{i}\mathrm{n}\mathrm{o}\mathrm{g}\mathrm{e}\mathrm{n}\mathrm{i}\mathrm{c}\,\mathrm{r}\mathrm{i}\mathrm{s}\mathrm{k} \left(\mathrm{C}\mathrm{R}\right)={\mathrm{C}\mathrm{D}\mathrm{I}}_{\mathrm{i}\mathrm{n}\mathrm{g}\mathrm{e}\mathrm{s}\mathrm{t}\mathrm{i}\mathrm{o}\mathrm{n}/\mathrm{d}\mathrm{e}\mathrm{r}\mathrm{m}\mathrm{a}\mathrm{l}}\times \mathrm{S}\mathrm{F}\times \mathrm{A}\mathrm{D}\mathrm{A}\mathrm{F}$$

SF is the slope factor for cancer for the studied HMs and ADAF denotes age-dependent adjustment factor (USEPA, 2004). The USEPA-guided acceptable CR range lies in the range of 10^−4^ to 10^−6^. Above this limit, any risk can led to serious health hazards.

### Geospatial and statistical analysis

The geostatistical analysis has been conducted in ArcGIS 10.3 software to interpolate the spatial distribution patterns of the HMs in the studied region. The Inverse Distance Weighting (IDW) model is a determining interpolation approach that allocates weights to points based on their distance, with closer points getting larger weightage. All the statistical analysis was performed in SPSS software.

### Speciation modelling using visual MINTEQ

Visual MINTEQ (version 4.0) was used to evaluate the chemical speciation of metals in water. The model predicted the distribution of dissolved metal species under aqueous conditions using measured physicochemical parameters. The metals considered were Pb, Ni, Cr, Cu, and Cd. Major cations (Na^+^, K^+^, Ca^2+^, Mg^2+^) and anions (SO_4_^2^⁻, PO_4_^3^⁻, Cl⁻, HCO_3_⁻) were included by inputting their measured concentrations (mg/L), which the model internally converted to molar units to enable speciation calculations. Measured pH and temperature were used to represent realistic environmental conditions. The system was assumed to be at thermodynamic equilibrium, using the default database constants provided in Visual MINTEQ.

## Results and discussion

### Chemical characteristics of surface water

Table 1 summarises the water quality characteristics of the analysed water samples and compares them with the WHO safe drinking water standards. The surface water in the study area was slightly alkaline, demonstrating pH values between 7.18 and 8.92, with a mean of 7.79 ± 0.41. Given that all samples fell within the allowable pH range of 7.0–8.5, the water is categorised as acceptable for consumption, as specified by both WHO (2022) and BIS (2012), and it is also suitable for irrigation as per Food and Agriculture Organization of the UN (FAO) (Ashie et al., 2024; FAO, 1985). The EC have a mean of 11,300 ± 4272.45 μS/cm and the wide variation indicated a complex local geochemical process. The EC of each surface water sample was above the accepted limits set by WHO (2022) and BIS (2012). The heightened levels of salinity in surface water are likely the result of human contributions, such as fertilizer inputs, local sewerage systems, and return irrigation flow. Earlier Brindha et al. (2014) mentioned an increased EC along a river flow owing to dumping of solid waste both from domestic and industrial origin, coincide with our results. In a recent study conducted by Lin et al. (2024) corroborated that industries often use high amount of chemicals and metals for manufacturing process which subsequently leads to a higher accumulation numerous salts in their discharge, ultimately leads to elevated EC level in the discharge outlet and corresponding waterbodies. Several studies reported that chemical fertilizer application and their subsequent export to canal water through runoff could be attributed to high EC of water (Huepa Briñez et al., 2024; Mudaly & Van der Laan, 2020). Moreover, concerning to the investigated area, the industrial activity could be the root cause of such high salinity in the collected water samples. Furthermore, the high amount of Cl^−^ in water supports this fact of increased EC (Guo et al., 2023). The TDS ranged between 1500 and 12,600 mg/L, with a mean of 6780 ± 2563.47 mg/L, exceeding permissible limit set by BIS (2012). Such high TDS are primarily caused by heightened dissolution Ca and Mg bearing minerals as previously reported by Bose et al. (2024). In case of ground water of Kolkata and its surrounding area, however, the industrial effluent discharge and dumping of solid waste is a significant cause of high TDS in surface water (Jamal & Ajmal, 2021). Dye industry plays a crucial role in contributing significant amount of total dissolved solids water as reported by Rajkumar and Nagan (2010). Since the sampled area is in a peri-urban area and the collected water samples were a part of a flowing water bodies like canal, thus, it may be possible that water samples were highly impacted with agricultural runoff. Such runoff/irrigation water are mostly contaminated with chemical fertilizer used for agriculture, and other agrochemicals which act as a wide spectrum non-point source of raising the salt concentration thereby increased the TDS level (Gomes et al., 2025;Kadam et al., 2021).

**Table 1:** Descriptive statistics of physicochemical attributes of collected water samples. Q3–Q1: Represents the inter quantile range, between 3rd quantile (Q3) and 1 st quantile (Q1) (*n* = 30)

Parameters	Maximum	Minimum	Mean	Std. dev	Q3–Q1
pH	8.92	7.18	7.79	0.41	7.99–7.55
EC (µS/cm)	21,000	2500	11,300	4272.45	13,475–8500
TDS (mg/L)	12,600	1500	6780	2563.47	8085–5100
TH (mg/L)	378.90	100.05	203.24	69.42	13.60–10.67
K^+^ (mg/L)	19.57	10.11	12.50	2.58	24.56–5.62
Na^+^ (mg/L)	49.89	0.92	17.87	12.81	48.33–23.19
Ca^2+^ (mg/L)	55.42	5.78	36.20	15.49	30.74–18.91
Mg^2+^ (mg/L)	64.98	10.10	27.49	13.12	125–85
Bi-carbonate (mg/L)	1305	130	738	273.46	871.25–581.25
Sulphur (mg/L)	37.81	6.84	18.67	8.21	23.58–12.45
Chlorine (mg/L)	433.10	294.65	366.24	39.01	401.15–338.13
Fluoride (mg/L)	1.22	0.28	0.57	0.24	0.80–0.37
Phosphorous (mg/L)	0.09	0.003	0.03	0.02	0.04–0.03
Ammoniacal nitrogen (mg/L)	1.54E − 04	1.9E − 05	6.8E − 05	4.5E − 05	0.01–0.005
Nitrate nitrogen (mg/L)	0.026	0.0013	0.012	0.006	0.00011–3.9E − 05
COD (mg/L)	356	256	310	24.69	0.019–0.008
BOD (mg/L)	74.28	27.59	48.32	12.00	327–296
Pb (mg/L)	0.51	0.12	0.23	0.10	58.36–38.46
Ni (mg/L)	0.47	0.03	0.19	0.13	5.07–0.95
Cr (mg/L)	2.16	0.22	0.97	0.54	43.17–22.14
Cu (mg/L)	0.17	0.011	0.060	0.038	0.48–0.08
Cd (mg/L)	0.017	0	0.004	0.005	240.34–157.43

The concentrations of major cations in the surface water samples are presented in Table 1. It is evident that calcium (Ca^2+^) is one of the dominating cations in the study area water samples, fluctuating between 5.78 and 55.42 mg/L (mean: 36.20 ± 15.49 mg/L); however, none of the samples exceeded the permissible limit set by WHO (2022). The observed concentration pattern is attributed to the weathering and dissolution of silicates minerals, especially Ca feldspars (Ganvir & Guhey, 2023). Also, evaporation of soil moisture can further concentrate calcium bearing minerals and may enhance leaching of calcium into water (Abanyie et al., 2023). Concentrations of magnesium (Mg^2+^) ranged between 10.10 and 64.98 mg/L (Mean: 27.49 ± 13.12 mg/L), with approximately 1% of samples being above the guideline of 50 mg/L (WHO, 2022). The elevated levels seen in a small portion of the samples can be attributed to natural weathering of magnesium-bearing minerals (such as dolomite and magnesite), but to some extent, discharges from wastewater, agriculture, and other anthropogenic influences may also contribute to increased Mg^2+^ concentrations in the surface water system (Nadjai et al., 2024). Overall, Ca^2+^ and Mg^2+^ presence in the water sample is majorly influenced by weathering of carbonate containing minerals, viz., calcite, gypsum, and cation exchange process in this scenario. Earlier Datta et al. (2021) found a similar distribution of Ca^2+^ and Mg^2+^ in the surface water collected around Kolkata Megacity area. Na^+^ and K^+^ values ranged from 0.92 to 49.89 and 10.11 to 19.57, with average of 17.87 and 12.50 mg/L, respectively. In general, low to moderate levels of sodium (Na^+^) and high levels of K^+^ in collected water samples can be explained by a complex geochemical interplay between natural weathering of minerals and important anthropogenic contributions such as fertilizer and industrial and municipal waste, with the dynamics of K^+^ often being more complicated than sodium (Kadam et al., 2021).

The anionic species present in the water samples is tabulated in Table 1, which clearly indicates that the dominance of HCO_3_⁻ and Cl⁻ ions followed by SO_4_^2⁻^ and F⁻. The presence of HCO_3_⁻ varied between 130 and 1305 mg/L across the study area which possibly attributed to the neutralisation of acidic waste by industries with lime (Ca(OH)_2_) or soda ash (Na_2_CO_3_) when acidic wastes are disposed of, creating leachates rich in bicarbonate likely enter to the nearby aquifers ultimately polluting the ground/surface water (Khan et al., 2013; Kumar et al., 2024). The distribution of Cl⁻ was very consistent among the samples with mean of 366.24 mg/L, exceeding the permissible limit set by WHO (2022). Such high levels of (Cl⁻) could be the consequence of untreated or partially treated effluent discharge associated with industries. According to Rajkumar and Nagan (2010), sodium chloride is the prime component of dye industry used for efficient fixing of dye in the fabric; consequently, waste/effluent disposed from these contaminates the surrounding water with high amount Cl⁻. Leachate from chloride-rich wastes and brine disposal may be another contributing factor resulting transport of Cl⁻ to surface and groundwater systems (Ratna et al., 2021). In addition to that, as the study area located in the South Bengal, which had a history of being inundated with marine or brackish water in the late quaternary time, therefore occurrence of entrapment of high Cl⁻ content during sedimentation which further modified in the confined aquifer, could be the major geological route of Cl⁻ in water of the sampled area (Banerjee & Sikdar, 2022). The source of S might be due to the oxidation of sulphide mineral (e.g. pyrite) in exposed soil or in waste piles generating sulphate which enters the water system through run off or leaching (Poot et al., 2024). The low P concentration suggests that phosphate-based compounds are not a significant part of dye-manufacturing industry effluents. Phosphate can also be eliminated in aquatic systems by adsorption and precipitation processes (Morshed et al., 2026). The study area is observed to have low to moderate F^−^ concentrations might be due to both natural and anthropogenic contributions. Naturally due to the dissolution of fluoride-bearing minerals such as fluorite and apatite in the aquifer (Li et al., 2025). In industrial areas, aside from F^−^ using industries such as aluminium, fertilizer, or some chemical plants, the contributing anthropogenic factor is minimal (Shaji et al., 2024). The ammoniacal nitrogen (NH_4_-N) concentrations in water samples was very low, ranging between 1.9E − 05 and 1.54E − 04 mg/L with a mean of 6.8E − 05 mg/L demonstrating low nitrogen loading across the study area. Concentrations of ammoniacal-nitrogen were much less than regulatory limit values indicating negligible risk of nitrogen compound-based pollution. Nitrate-nitrogen (NO_3_⁻-N) concentrations ranged from 0.0013 to 0.026 mg/L average of 0.012 mg/L and remained at trace levels making the water quality within WHO (2022) standards.

Water sample analysed from the study site’s location revealed high COD and moderate BOD, with an average COD of 310 mg/L (range 256 to 356 mg/L) and BOD of 48.32 mg/L (27.59–74.29 mg/L) (Table 1). The relatively high levels of COD indicate that a significant quantity of biodegradable and non-biodegradable organic matter is present (likely stemming from industrial discharge, possible process waste, and organic-rich effluent) (Lv et al., 2024). Conversely, moderate levels of BOD suggest that either a substantial quantity of the organic material is resistant to microbial breakdown, or that the activity of microorganisms is impaired by high salinity, toxic or inhibitory materials present in the effluent (Elnabi et al., 2025). The average BOD/COD ratio (~ 0.16) similarly indicates the prevalence of recalcitrant organic compounds, reflecting the influence of industrial domination rather than domestic or biodegradable waste inputs. Therefore, the reach of organic pollution to the water likely affects the health of aquatic ecosystems and the management of water quality.

### Heavy metals

The total concentration of heavy metals in water samples is depicted in Table 1. The total metal content (mg/L) in the water samples is as follows: Cr = 0.22–2.16; Ni = 0.03–0.47; Cu = 0.011–0.17; Pb = 0.12–0.51; and Cd = 0–0.017. For Cr, Pb, Ni, Cu, and Cd, mean heavy metal concentrations in the study sites follow the order: Cr (0.97 mg/L) > Pb (0.23 mg/L) > Ni (0.19 mg/L) > Cu (0.060 mg/L) > Cd (0.004 mg/L). Except for Cu, all values were higher than the permissible limit of the WHO (2022). Increased Cr, Pb, and Ni concentrations are primarily related to discharges from the dye manufacturing unit along with other metallurgical processes (Singare & Dhabarde, 2014). Chromium is widely utilised in dye and textile industries for the processes of colouring and mordanting, leading to high levels of Cr in wastewater (Gayathri et al., 2025). Ni and Pb can either stem from catalysts, pigments, and metal-containing dyes as well as corrosion from the machinery and pipes used in industrial processes. The continuous arrival of such metal-laden effluent to surface waters, especially when inadequately treated or improperly disposed of accounts for the historical accumulation of these metals in the water bodies, demonstrating the persistent anthropogenic impacts of industrial processes in the area (Agrawal et al., 2021). The relatively low concentrations of copper (Cu) at the study site may either be a result of the low amounts of Cu used in dye production in the area, or the limited release of Cu due to its strong retention by soils and sediments from either adsorption onto organic matter or clay minerals. Furthermore, copper can precipitate as insoluble compounds depending on the pH and redox potential, limiting mobility through the water column (Oladimeji et al., 2024). The presence of Cd, which, even at a low yet threatening concentration, is observed in this area due to anthropogenic activities related to industry or as an impurity in phosphorous-based dyes and fertilizers. The release of Cd into aqueous systems could also potentially leach from small-scale effluent from industrial wastewater, sludge, or contaminated soils (Idrees et al., 2018).

### Spatial distribution of HMs

**Fig. 2:**
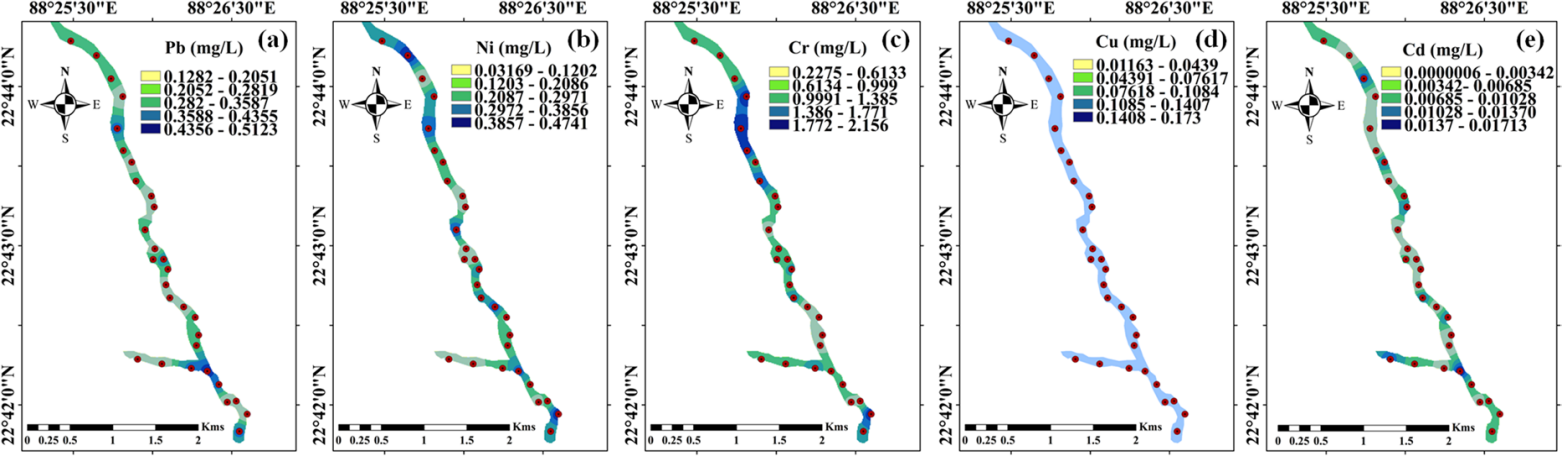
IDW-based spatial distribution maps illustrating the concentrations of Pb (a), Ni (b), Cr (c), Cu (d), and Cd (e) in collected water samples from the study area

Figure 2 demonstrates the spatial distribution (IDW interpolation) of the studied heavy metals (Cd, Cr, Ni, Cu, Pb) in the study area. The IDW maps indicate significantly higher concentrations of Ni and Cr in the downstream section compared to the upstream. Pb also shows elevated concentrations in the downstream region, although the gradient is more gradual. Cd and Cu are distributed throughout the stream, showing relatively lower concentration ranges, with a localised hotspot for Cu in the mid-to-lower reaches. Overall, there is a clear downstream accumulation trend for multiple heavy metals, suggesting that the major contamination sources are concentrated in the lower portion of the canal.

Spatial correlation analysis was further conducted through variogram modelling, and the comparative performance of the spherical, exponential, Gaussian, and Matern models is presented in Supplementary Table 2. Among these, the Gaussian model provided the best fit for most metals based on the lowest RSS values (Ni: 0.00839; Pb: 0.00628; Cu: 0.000455), whereas Cr and Cd were best described by the exponential (Cr: 2.87) and spherical (Cd: 3.9E − 08) models, respectively. The range values (expressed in kilometres after UTM projection) suggest that spatial dependence persists over several kilometres before semi-variance stabilises, indicating spatial independence beyond these distances (Karami et al., 2013). Cd shows moderate spatial autocorrelation, with concentrations varying smoothly over distances of approximately 6–17 km. Cr exhibits clear spatial dependence over moderate ranges (15–28 km). Ni shows strong spatial autocorrelation, with relatively large ranges (10–28 km). Pb displays the second-largest spatial range (up to 46 km), indicating very strong long-distance spatial influence. Cu shows the greatest spatial continuity, with extremely large ranges (up to 54 km), suggesting highly uniform and persistent spatial dispersion across the study area. The generally low nugget-to-sill ratios further confirm that spatial variability is primarily structural rather than attributed to measurement errors or microscale fluctuations. These variogram-based insights complement the IDW interpolation results.

### Pollution indices

#### Single factor pollution index (P_i_)

The *P*_*i*_ of heavy metals (HMs) was determined using Eq. (4). The average *P*_*i*_ values of Pb, Ni, Cr, Cu, and Cd in the surface water samples were 23.07, 10.90, 18.82, 0.04, and 1.46, respectively (Supplementary Table 3). Considering the type of pollution in surface water, the water was seriously polluted with Pb, Ni, Cr, and Cd. The *P*_*i*_ value of Cu is less than 1 and indicates that the surface water is not polluted with Cu. Therefore, the area is heavily polluted by Pb, Ni, Cr, and Cd.

#### Degree of contamination (C_d_)

The mean *C*_*d*_ of this study area is 48.89 (Supplementary Table 3), with minimum and maximum values of 19.28 and 93.40, respectively. All the samples from the study area were rated above the highly polluted category with *C*_*d*_ values above 3. Edet et al. (2002) proposed a modified three-layer classification based on the *C*_*d*_ value by using a multiple of the mean *C*_*d*_ value. Therefore, the modified groups are as follows: (*C*_*d*_ < 20): Low, (*C*_*d*_ 20–40): Medium, and (*C*_*d*_ > 40): High. Based upon this categorisation, 3%, 36%, and 61% water samples were considered as low polluted, medium or moderately polluted, and highly polluted respectively (Supplementary Table 4).

#### Heavy metal evaluation index (HEI)

To assess the overall quality and evaluate the trend in relation to the permissible limit of heavy metals, Singh et al. (2017) proposed HEI. The mean HEI calculated for this study area was 53.65, with minimum and maximum values of 24.29 and 98.41 (Supplementary Table 3). 100% computed HEI values in the study area fell within greater than 30, indicating that the samples exceeded the extremely elevated metal evaluation index. Following Edet et al. (2002) approach, approximately 36.67% and 63.33% of the samples were categorised as moderate and elevated pollution classes based on the modified HEI classifications (Supplementary Table 4). HEI had a strong positive relationship with Pb, Ni, and Cr. Thus, we can infer that Pb, Ni, and Cr were the major heavy metals contributing to the contamination of the aqueous media of the study area.

#### Heavy metal pollution index (HPI)

The calculated mean for the HPI of the studied water samples was 337.73, with minimum and maximum of 75.83 and 762.09 respectively (Supplementary Table 3). All calculated HPI values were high indicating the samples were more than unsuitable for usage. Edet et al. (2002) proposed classifying HPI values into three pollution categories: low, medium, and high. According to the calculated HPI value, 20%, 50%, and 30% of the samples comes under low, medium, and high pollution classes respectively. This confirms that water samples are completely unsuitable for human consumption (Supplementary Table 4).

#### Water pollution index

The Water Pollution Index (WPI) calculated for 30 surface water samples from the industrial area is between 1.51 and 5.27, with a mean value of 2.86 (Supplementary Table 3). Based on the classification developed by several researchers (Ganaie et al., 2023; Hossain & Patra, 2020a, b), when WPI < 0.5 indicates good water quality, WPI between 0.5 and 0.75 indicates moderately polluted water and if WPI between 0.75 and 1; highly polluted water, when WPI > 1. All the samples recorded a value of WPI > 1 thus demonstrating that water samples are highly polluted owing to its industrial association/intervention (Supplementary Table 4). Moreover, values greater than 1 display serious pollution likely due to unregulated industrial discharges, heavy metal leaching, and the dumping of effluents nearby. Such higher values of WPI emphasise the collective contributions of various pollutants, rather than influence of individual parameters. The mean overall WPI of 2.86 places the water quality overall in a poor category and unfit for human use, which indicates the requirements of appropriate sanitising treatments prior to use. Most importantly areas close to dye manufacturing and small-scale industrial units had the highest WPI which indicates a strong anthropogenic influence on water quality deterioration.

#### Evaluation of irrigation indices

The irrigation suitability of the collected water samples was assessed using three important criteria: Sodium Adsorption Ratio (SAR), Sodium Percentage (Na%), and Kelly’s Ratio (KR). These criteria indicate the possible effects of sodium and other cations on soil permeability and crop productivity. The SAR values of the evaluated water samples ranged from 0.16 to 8.66, with a mean of 3.34 (Table 2). According to the US Salinity Laboratory classification, all samples belong to “Excellent” (< 10 SAR) category, which indicates that risk from sodium in the irrigation water is minimal. Therefore, no negative impact on soil structure due to sodium accumulation is anticipated. The 100% suitability suggests that the water is safe for irrigation based on sodium hazard. The average Na% value was 32.69, with a range of 12.73 to 56.30. Ninety-four percent of the samples were classified as “Good” (Na% < 50) and 6% as “Unsuitable” (Na% > 50) for irrigation usage according to the Wilcox classification (Table 2). Reduced infiltration rates and soil dispersion can result from irrigation water with a high sodium content. However, the total sodium risk is regarded as minimal because most of the samples showed Na% values within the recommended range. Only a small number of samples showed possible problems that would necessitate adding soil additives like gypsum or blending with high-quality water. The KR values ranged from 0.014 to 0.96, with a mean value of 0.32 (Table 2). All of the samples have been classified as appropriate for irrigation because their KR values were less than 1. Excess sodium over calcium and magnesium is indicated by a KR value greater than 1, which renders the water unfit for irrigation. The low KR values found in every sample indicate to the dominance of divalent cations over sodium, which preserves soil permeability and avoids sodicity issues.

**Table 2:** Evaluation of the suitability of water samples for irrigation by analysing major irrigation indices

Irrigation indices	Mean	Range (min–max)	Suitability ranges	Samples in each class
Sodium adsorption ratio (SAR)	3.34	0.16–8.66	Excellent <10	100%
			Good 10–18	
			Doubtful 18–26	
			Unsuitable >26	
Na%	32.69	12.73–56.30	Good <50	94%
			Unsuitable >50	6%
KR	0.32	0.014–0.96	Unsuitable >1	100%
			Suitable <1	

#### Visual MinteQ and speciation

The chemical forms of metals are numerous, and their speciation will have a significant impact on the geochemical processes associated with a metal, including the mobility, solubility, ability to precipitate, and its bioavailability in aqueous system (Fytianos, 2001). Table 3 shows how the various forms of metals were distributed within the water samples. Speciation studies were performed in this study to confirm or validate results of the physicochemical analysis of the water samples. The speciation of cadmium in the water samples (Table 3) indicates Cd^2+^ as the predominating species at an average of 52.35% (27.57–61.72%). Cadmium exists primarily in the free ionic form, increasing its solubility and mobility at the system’s increasingly acidic to near-neutral condition. The hydrolysed species (CdOH^+^) were minimal (< 0.50%), which indicates limited hydrolysis of Cd^2+^ was occurring since pH and alkalinity were extremely low for those precipitation reactions to occur. Chloride complexes, specifically the isolated complex CdCl^+^, accounted for most of the remaining fractions (32.10%), which indicates that chloride has more of a controlling influence on cadmium speciation and related transport (Kubier et al., 2019). Very little contribution was made by the aqueous species CdCl_2_ (1.06%) or cadmium bicarbonate (1.48%), which indicates a low contribution of carbonate complexation mainly due to the competing influence of chloride. Thus, cadmium in this study occurred predominantly in ionic dissolved and chloride-bound forms, which favours mobility compared to precipitation. Very little hydroxyl or carbonate species indicate that there is little to no formation of Cd(OH)_2_ or CdCO_3_ species. This pattern is consistent with the literature on low-alkalinity waters, where the Cd^2+^ and CdCl⁺ are the dominant species and are likely to be more mobile in the system and bioavailable than those associated with hydroxyl and carbonate complexation (Kubier et al., 2019).

**Table 3:** Percentage distribution of heavy metal species in the collected water samples from Visual MINTEQ Modelling

Range	Cd					Pb					Ni					Cr			Cu			
	Cd^2+^	CdOH^+^	CdCl^+^	CdCl_2_	CdHCO^3+^	Pb^+2^	PbOH^+^	PbCl^+^	PbCO_3_	PbHCO^3+^	Ni^2+^	NiOH^+^	NiSO_4_	NiCO_3_	NiHCO^3+^	CrO_4_^−2^	HCrO_4_^−^	CaCrO_4_	Cu^2+^	CuOH^+^	CuCO_3_	CuHCO^3+^
Max	61.72	1.93	39.45	1.50	2.15	29.02	10.70	6.78	65.72	72.14	90.92	3.23	1.31	64.48	12.11	96.02	12.65	21.47	26.82	9.40	74.59	1.26
Min	27.57	0.05	18.91	0.71	1.10	0.29	4.25	0.01	13.21	0.44	25.68	0.12	0.14	0.01	4.52	69.17	0.22	3.31	0.16	3.00	61.66	0.02
Mean	52.35	0.50	32.10	1.06	1.48	13.00	7.18	3.12	39.47	31.31	63.15	1.29	0.66	26.26	7.54	77.43	6.31	16.02	11.67	6.57	67.94	0.63

The speciation results for lead (Pb) (Table 3) indicated that carbonate complexes dominate its distribution. PbHCO_3_⁺ and PbCO_3_ (aq) were the major fractions, with average values of 31.31% and 39.47%, respectively, indicating that Pb interacts strongly with carbonate species at the prevailing geochemical conditions, and therefore, speciation generally promotes lead stability in solution, while decreasing the number of free ions (Kubier et al., 2019). Pb^2^⁺ was a mean of 13.00% (0.29–29.02%) which indicates moderate mobility potential in the aqueous phase. Hydrolysed species (PbOH⁺) contributed a modest 7.18% and indicate some degree of hydrolysis at the mildly alkaline conditions. PbCl^+^ occurred in minimal concentrations (mean = 3.12%), indicating limited chloride complexation. Overall, the dominance of carbonate species indicated that Pb was in the dissolved carbonate complex form, rather than as free ionic species, which may decrease the immediate mobility of Pb, while increasing persistence in solution. This distribution is consistent with environmental conditions of moderate to high alkalinity, in which carbonate complexation will dominate Pb speciation and geochemical behaviour.

The distribution of Ni speciation in the water samples (Table 3) indicates that the free divalent ion (Ni^2^⁺) is the dominant species (average 63.15% (25.68–90.92%)), demonstrating that Ni is mostly present in its ionic form, which enhances its mobility and bioavailability in the water column, especially at low to neutral pH (Town & van Leeuwen, 2022). Hydrolysed species (NiOH^+^) were low (mean = 1.29%), indicating minimal hydrolysis, and sulphate complexes (NiSO_4_ (aq)) were very low (0.66%). Co-precipitated carbonate complexes were moderate, with NiCO_3_ (aq) and NiHCO_3_^+^ accounting for 26.26% and 7.54% on average, indicative of some interaction with carbonate species, likely under mildly alkaline conditions. As evidenced by the predominance of Ni^2^⁺, soluble and mobile nickel remains soluble, while carbonate species do not seem to precipitate or lead to significant immobilisation of Ni; however, pH and alkalinity modify nickel speciation (Cieślik et al., 2025).

The clear pre-eminence of the chromate ion (CrO_4_^2−^) over bichromate (HCrO_4_^−^) in all the samples suggests that the water is alkaline (pH > 7), which facilitates migration of this anionic contaminant and increases the potential for it to be transported more broadly and delivered to the ecosystem (Table 3). Nevertheless, there is some amelioration of risk from other natural attenuation processes based on the abundance of solid calcium chromate (CaCrO_4_). The direct implication here is that the dissolved Cr(VI) concentration is held in check by precipitation, an important process by which toxic metal is immobilised and made less bioavailable to living systems (McNeill et al., 2012). Accordingly, the overall threat to the environment posed by chromium within this system relates to balancing the mobility of the dissolved chromate against its potential immobilisation based on the precipitation with available calcium.

Dissolved Cu was mainly associated to the neutral carbonate complex, CuCO_3_ (aq), which made up a mean of 67.94% of the species distribution (61.66%–74.59% range) (Table 3). The free, bioavailable Cu^2+^ was proportionally variable but consistently lower than the complexed copper (mean 11.67% and range from 0.16% to 26.82%) and was highly inversely correlated with pH. The hydroxo-complex was the second most stable form, with an average of 6.57% and the bicarbonate complex was negligible throughout (mean 0.63%). This consistent observation shows that in this system, solubility, mobility, and potential bioavailability of Cu are primarily controlled through copper-carbonate complexation, thus lowering the concentration of the more reactive and toxic free cationic Cu^2+^ (Hu et al., 2017).

### Health risk assessment

#### Non-carcinogenic health risk

In this study, a health risk assessment was performed to evaluate the level of adverse effects of hazardous elements among the populations via the dermal and ingestion pathway. An HQ value greater than 1 suggests a potential for adverse non-carcinogenic effects while values less than 1 indicate low risk. The maximum HQ values for adults for Pb, Ni, Cr, Cu, and Cd via skin contact were 2.93E − 06, 4.75E − 07, 1.44E − 05, 9.34E − 08, and 3.43E − 07 respectively, and the average HQ values were 1.32E − 06, 1.93E − 07, 6.47E − 06, 3.26E − 08, and 8.78E − 08 respectively, which were < 1 or insignificant non-carcinogenic risk to adults through dermal exposure (Table 4). For children, the maximum HQ values for Pb, Ni, Cr, Cu, and Cd via skin contact were 4.47E − 05, 7.23E − 06, 2.19E − 04, 1.42E − 06, and 5.23E − 06 respectively, and the average HQ values were 2.01E − 05, 2.94E − 06, 9.87E − 05, 4.97E − 07, and 1.34E − 06 respectively (Table 4). Particularly, the HQ for children via skin contact did not exceed the safe limit (HQ > 1), suggesting that children are situated on a safe zone. This elevated risk can be primarily attributed to their higher surface area to weight ratio, and they have more direct exposure to contaminated water and soil.

**Table 4:** Non-carcinogenic risk generated from the heavy metals in the sampled area

Heavy metals	HQ ingestion adult			HQ ingestion child			HQ dermal adult			HQ dermal child		
	Maximum	Minimum	Mean	Maximum	Minimum	Mean	Maximum	Minimum	Mean	Maximum	Minimum	Mean
Pb	5.14E + 00	1.29E + 00	2.31E + 00	7.84E + 00	1.96E + 00	3.53E + 00	2.93E − 06	7.33E − 07	1.32E − 06	4.47E − 05	1.12E − 05	2.01E − 05
Ni	8.33E − 01	5.55E − 02	3.39E − 01	1.27E + 00	8.46E − 02	5.16E − 01	4.75E − 07	3.16E − 08	1.93E − 07	7.23E − 06	4.82E − 07	2.94E − 06
Cr	2.53E + 01	2.66E + 00	1.14E + 01	3.85E + 01	4.05E + 00	1.73E + 01	1.44E − 05	1.52E − 06	6.47E − 06	2.19E − 04	2.31E − 05	9.87E − 05
Cu	1.64E − 01	1.09E − 02	5.72E − 02	2.50E − 01	1.66E − 02	8.71E − 02	9.34E − 08	6.22E − 09	3.26E − 08	1.42E − 06	9.49E − 08	4.97E − 07
Cd	6.03E − 01	0.00E + 00	1.54E − 01	9.18E − 01	0.00E + 00	2.35E − 01	3.43E − 07	0.00E + 00	8.78E − 08	5.23E − 06	0.00E + 00	1.34E − 06

For the ingestion portion, the mean HQ values for adults were 2.31E + 00 (Pb), 3.39E − 01 (Ni), 1.14E + 01 (Cr), 5.72E − 02 (Cu), and 1.54E − 01 (Cd) (Table 4). Additionally, HQ values for Pb and Cr were greater than 1, suggesting a potential non-carcinogenic risk for oral exposure to these metals for adults. For children, the mean HQ values for ingestion were 3.53E + 00 (Pb), 5.16E − 01 (Ni), 1.73E + 01 (Cr), 8.71E − 02 (Cu), and 2.35E − 01 (Cd) (Table 4). Children also had considerably higher HQ values than adults, with Pb and Cr exceeding the daily allowable values. This means that children may be more sensitive to the non-carcinogenic effects of these metals through ingestion compared to adults, as young children have lower body weight and consume more water than adults relative to total body weight.

#### Carcinogenic health risk

The potential carcinogenic risk assessment of the study area was determined by evaluating all the metal through dermal and ingestion exposure for adults and children. The maximum, minimum, and mean for the carcinogenic risk assessment and sum CR are summarised and shown in Table 5. The USEPA risk assessment level guideline for the carcinogenic risk indicates that the below values of 1 × 10^–6^ are not likely to have significant impact on human health, the values between 1 × 10^–6^ and 1 × 10^–4^ are acceptable or tolerable, and the above values of 1 × 10^–4^ are unacceptable and most harmful for human health (USEPA, 2012). In the 30 surface water samples, all the CR values for the metals analysed in adults were all below the level of 1 × 10^–6^ representing very minimal carcinogenic risk through dermal exposure. Specifically, the CR values for Pb ranged from 2.18E − 10 to 8.72E − 10, for Ni from 5.76E − 10 to 8.64E − 09, for Cr from 3.46E − 08 to 3.29E − 07, for Cu from 5.98E − 09 to 8.98E − 08, and for Cd from 0.00 to 1.31E − 10. Among the metals considered, Cr showed significantly higher CR values than the other metals, although still in the acceptable range. For children, the CR values were Pb 3.32E − 09 to 1.33E − 08, Ni 8.78E − 09 to 1.32E − 07, Cr 7.58E − 07 to 7.20E − 06, Cu 1.06E − 08 to 1.59E − 07, and Cd 0 to 1.99E − 09. Among the analytes, Cr represented the highest CR values, and several samples were at or slightly above 1 × 10^–6^, which is the lower limit of the acceptable risk range would indicate that chromium is primarily responsible for dermal carcinogenic risk in children due to its higher dermal absorption and carcinogenic potential, particularly for hexavalent chromium species.

**Table 5:** Carcinogenic risk generated from the heavy metals in the sampled area

Heavy metals	CR ingestion adult			CR ingestion child			CR dermal adult			CR dermal child		
	Maximum	Minimum	Mean	Maximum	Minimum	Mean	Maximum	Minimum	Mean	Maximum	Minimum	Mean
Pb	1.53E − 03	3.82E − 04	6.88E − 04	2.33E − 03	5.83E − 04	1.05E − 03	8.72E − 10	2.18E − 10	3.92E − 10	1.33E − 08	3.32E − 09	5.98E − 09
Ni	1.52E − 02	1.01E − 03	6.16E − 03	2.31E − 02	1.54E − 03	9.39E − 03	8.64E − 09	5.76E − 10	3.51E − 09	1.32E − 07	8.78E − 09	5.35E − 08
Cr	5.77E − 02	6.08E − 03	2.60E − 02	1.26E + 01	1.33E + 00	5.68E + 00	3.29E − 07	3.46E − 08	1.48E − 07	7.20E − 06	7.58E − 07	3.24E − 06
Cu	1.57E − 02	1.05E − 03	5.49E − 03	2.78E − 01	1.86E − 02	9.72E − 02	8.98E − 08	5.98E − 09	3.13E − 08	1.59E − 07	1.06E − 08	5.54E − 08
Cd	2.29E − 04	0.00E + 00	5.85E − 05	3.49E − 04	0.00E + 00	8.92E − 05	1.31E − 10	0.00E + 00	3.34E − 11	1.99E − 09	0.00E + 00	5.08E − 10

The calculated CR values for ingestion by adults from the analysed samples varied from 3.82E − 04 to 1.53E − 03 for Pb, 1.01E − 03 to 1.52E − 02 for Ni, 6.08E − 03 to 5.77E − 02 for Cr, 1.05E − 03 to 1.57E − 02 for Cu, and 0 to 2.29E − 04 for Cd (Table 5). Across all samples, the average CR values for Pb, Ni, Cr, Cu, and Cd were 6.88E − 04, 6.16E − 03, 2.60E − 02, 5.49E − 03, and 5.85E − 05 respectively. The highest CR value among all the metals was observed for Cr, followed by Ni and Cu. The CR values of  ingestion for  children for Pb, Ni, Cr, Cu, and Cd were calculated and presented in Table 5. The CR values had a large variance among the metals, with Cr having the highest CR values across all sampling sites (mean: 5.68E + 00), followed by Ni, Pb, Cu, and Cd. For Cr, all calculated CR values for children were significantly higher than the acceptable risk range by USEPA (2012) which indicates a high potential carcinogenic risk.

## Conclusion

Hydrogeochemical assessment demonstrate that surface water quality deterioration in studied industrial area was primarily driven by natural geological processes and further aggravated due to anthropogenic activities. EC and TDS levels exceeding the WHO limits, indicating mineralised and saline water quality impacted by industrial sewage disposal, irrigation return flow and agrochemical leaching. Ca^2+^ and HCO_3_⁻ ions concentrated to a high degree and indicated mineral weathering processes. Cl⁻ and SO_4_^2^⁻ concentrated to a high degree from continuous intrusion of industrial effluents. High COD and low BOD/COD ratios indicate resistant industrial organics and heavy organic pollution. Heavy metal concentration crossed the international water quality standards due to metal processing industries. The pollution indices *P*_*i*_, *C*_*d*_, HEI, HPI, and WPI inferred that the water is severely polluted. Despite fit for irrigation as per SAR, Na%, and KR indices the HM-driven health risk persists significantly. Speciation analysis depicted that Cd^2^⁺ and Ni^2^⁺ exists as free ions, enhancing greater mobility and its biological uptake. Pb and Cu were mostly associated with carbonate complexes, while Cr(VI) occurred as mobile chromate species, projecting substantial risk to the environment. Human health risk assessment revealed that ingestion was the most detrimental route of HM-exposure, indicating Cr and Pb being the hazardous for both adults and children, while the carcinogenic risk predominantly posed by Cr exceeded USEPA acceptable limits. Overall, the findings of the study emphasise the urgent need for a systematic effluent control, sustained water monitoring, and targeted remediation for protecting public and environmental health.

## Supplementary Information

Below is the link to the electronic supplementary material.

ESM 1 (DOCX 24.5 KB)

## Data Availability

The data will be made available on request.
