# Supplementary material for: Comprehensive evaluation of surface water quality: heavy metals, speciation, and human health risks in an industrial region of West Bengal, India
Source: Environ Monit Assess. 2026 May 23;198(6):644. doi: 10.1007/s10661-026-15424-9 (PMC13198492; doi:10.1007/s10661-026-15424-9)
Supplement: Supplementary file 1 — (DOCX 24.5 KB) [file 10661_2026_15424_MOESM1_ESM.docx]

**Supplementary Information**

**Comprehensive Evaluation of Surface Water Quality: Heavy Metals, Speciation, and Human Health Risks in an Industrial Region of West Bengal, India**

Gourav Mondal^1^, Riddhi Basu^1^, Sumit Kumar^1^, Shreya Chakraborty^1^, Ambika Barman^1^, Kasturi Charan^1^, Jajati Mandal^2*^, Pradip Bhattacharyya^1*^

^1^*Agricultural and Ecological Research Unit, Indian Statistical Institute, Giridih, Jharkhand, India, 815301*

^2^*School of Sciences, University of Salford, Engineering & Environment, Manchester M5 4WT, UK*

Author e-mail addresses:

Gourav Mondal: [gouravmondal.241992@gmail.com](mailto:gouravmondal.241992@gmail.com)

Riddhi Basu: [basuriddhi099@gmail.com](mailto:basuriddhi099@gmail.com)

Sumit Kumar: [Sumit29sep@gmail.com](mailto:Sumit29sep@gmail.com)

Shreya Chakrabarty: [shreyadona94@gmail.com](mailto:shreyadona94@gmail.com)

Ambika Barman: [ambikabarman864@gmail.com](mailto:ambikabarman864@gmail.com)

Kasturi Charan: [kasturicharan13@gmail.com](mailto:kasturicharan13@gmail.com)

*^*^Corresponding authors:* [*J.Mandal2@salford.ac.uk*](mailto:J.Mandal2@salford.ac.uk) *,* [*pradip.bhattacharyya@gmail.com*](mailto:pradip.bhattacharyya@gmail.com)

**Supplementary Table 1.** Values of recovery analysis for water samples.

| **HMs** | **SRM 2710**  **(mg/ kg)** | **AAS measured (mg/ kg)** | **Recovery (%)** |
| --- | --- | --- | --- |
| **Cr** | 17 | 17.4 | 97.7 |
| **Ni** | 10 | 10.18 | 98.23 |
| **Pb** | 5104 | 5189 | 98.36 |
| **Cu** | 2700 | 2727 | 99 |
| **Cd** | 19 | 19.12 | 99.37 |

**Supplementary Table 2.** Summary of semi-variogram model parameters including nugget, sill, range (km), and residual sum of squares (RSS) used to evaluate spatial variability and model performance.

| **HMs** | **Model** | **Nugget** | **Sill** | **Range (Km)** | **RSS** |
| --- | --- | --- | --- | --- | --- |
| **Cd** | Spherical | 1.52E-05 | 3.01E-05 | 16.95096 | 3.91E-08 |
| **Cd** | Exponential | 0 | 0 | 53.781 | 1.38E-06 |
| **Cd** | Gaussian | 9.17E-06 | 3.35E-05 | 6.436024 | 4.58E-08 |
| **Cd** | Matern | 0 | 0 | 53.781 | 1.38E-06 |
| **Cr** | Spherical | 0 | 0.232941 | 27.95536 | 2.691815 |
| **Cr** | Exponential | 0 | 0.259021 | 15.01097 | 2.877324 |
| **Cr** | Gaussian | 0 | 0.243578 | 21.06537 | 5.650121 |
| **Cr** | Matern | 0 | 0.259021 | 15.01097 | 2.877324 |
| **Ni** | Spherical | 0 | 0.018767 | 28.25789 | 0.008356 |
| **Ni** | Exponential | 0 | 0.018553 | 9.792452 | 0.011787 |
| **Ni** | Gaussian | 0 | 0.018722 | 13.202 | 0.008391 |
| **Ni** | Matern | 0 | 0.018553 | 9.792454 | 0.011787 |
| **Pb** | Spherical | 0 | 0.014571 | 46.20049 | 0.007335 |
| **Pb** | Exponential | 0 | 0.015141 | 21.72671 | 0.01076 |
| **Pb** | Gaussian | 0 | 0.014736 | 20.71896 | 0.006283 |
| **Pb** | Matern | 0 | 0.015141 | 21.72672 | 0.01076 |
| **Cu** | Spherical | 0.001946 | 0.001946 | 53.781 | 0.000504 |
| **Cu** | Exponential | 0.001934 | 0.001934 | 53.781 | 0.000495 |
| **Cu** | Gaussian | 0.001881 | 0.001881 | 53.781 | 0.000455 |
| **Cu** | Matern | 0.00189 | 0.00189 | 53.781 | 0.000461 |

**Supplementary Table 3.** Single factor pollution indexes for determination of water quality of the area.

| **Pollution indexes** | **Mean** | **Range (Min-Max)** |
| --- | --- | --- |
| **P_i_ (Pb)** | 23.07 | 12.82-51.28 |
| **P_i_ (Ni)** | 10.90 | 1.58-45.45 |
| **P_i_ (Cr)** | 18.82 | 0.46-43.18 |
| **P_i_ (Cu)** | 0.04 | 0.007-0.11 |
| **P_i_ (Cd)** | 1.46 | 0-5.72 |
| **C_d_** | 48.89 | 19.28-93.40 |
| **HEI** | 53.65 | 24.29-98.41 |
| **HPI** | 337.73 | 75.83-762.09 |
| **WPI** | 2.86 | 1.51-5.27 |

**Supplementary Table 4.** Evaluation of different pollution indexes for the determination of water quality.

| **Pollution indexes** | **Degree of contamination** | **Samples in each class** |
| --- | --- | --- |
| **C_d_** | Low < 20 | 3% |
|  | Medium 20-40 | 36% |
|  | High > 40 | 61% |
| **P_i_** | Low 1-2 (Type ii) | 16% for Ni, 26.66% for Cd |
|  | Slight 2-3 (Type iii) | 20% for Cd |
|  | Moderate 3-5 (Type iv) | 16% for Pb, 10% for Cr |
|  | High > 5 (Type v) | 68% for Pb, 90% for Cr, 3.33% for Cd |
| **HPI** | Low < 200 | 20% |
|  | Moderate 200-400 | 50.00% |
|  | High > 400 | 30% |
| **HEI** | Low < 30 | 0% |
|  | Moderate 30-60 | 63.33% |
|  | High > 60 | 36.67% |
| **WPI** | Excellent < 0.50 |  |
|  | Good 0.50-0.75 |  |
|  | Moderate pollution 0.75-1 |  |
|  | High pollution > 1.00 | 100% |
